# The Burden of Atherosclerotic Cardiovascular Disease Risk Factors Among Adults Living With HIV in the Ashanti Region: A Cross-Sectional Study in Kumasi

**DOI:** 10.7759/cureus.97635

**Published:** 2025-11-24

**Authors:** Manasseh B Wireko, Patrick W Narkwa, Emmanuel A Ntim, Lorraine Sallah, Betty Norman, Jacobus Hendricks, Kweku Bedu-Addo, Marlise V Staden, John A Larbi, Isaac K Owusu

**Affiliations:** 1 Biological Science Education, Akenten Appiah-Menka University of Skills Training and Entrepreneurial Development, Kumasi, GHA; 2 Theoretical and Applied Biology, Kwame Nkrumah University of Science and Technology (KNUST), Kumasi, GHA; 3 Clinical Microbiology, Kwame Nkrumah University of Science and Technology (KNUST), Kumasi, GHA; 4 Physiology, School of Medical Sciences, Kwame Nkrumah University of Science and Technology (KNUST), Kumasi, GHA; 5 Physiology, Kwame Nkrumah University of Science and Technology (KNUST), Kumasi, GHA; 6 Internal Medicine, Kwame Nkrumah University of Science and Technology (KNUST), Kumasi, GHA; 7 Internal Medicine, Komfo Anokye Teaching Hospital, Kumasi, GHA; 8 Physiology and Environmental Health, University of Limpopo, Limpopo, ZAF; 9 Medicine, Kwame Nkrumah University of Science and Technology (KNUST), Kumasi, GHA

**Keywords:** atherosclerotic cardiovascular disease (ascvd), diabetes mellitus, dyslipidemia, highly active antiretroviral therapy (haart), hypertension, metabolic syndrome, obesity, sub-saharan africa

## Abstract

Background

The burden of atherosclerotic cardiovascular disease (ASCVD) in sub-Saharan Africa (SSA) is increasing rapidly. Risk factor assessment is necessary for specific and strategic interventions to curb the high ASCVD burden. Evidence suggests that people living with HIV (PLWH) are at greater risk of ASCVD compared to HIV-negative individuals, as they spend many years on highly active antiretroviral therapy (HAART). This study therefore sought to explore the prevalence of ASCVD risk factors among adults receiving HAART in the Ashanti Region.

Methods

A hospital-based cross-sectional study design and systematic sampling were used to recruit 254 PLWH. Anthropometric measurements and lifestyle factors, including alcohol consumption, were assessed using the WHO STEPS protocol. Blood pressure measurements and blood samples were taken to determine participants’ blood sugar levels (HbA1c) and lipid profiles. Participants’ HAART history was obtained from their medical record cards. Chi-square tests were used to assess differences in categorical ASCVD risk outcomes, while one-way ANOVA was used to determine differences in mean values of continuous risk factors.

Results

Most participants were female (85.43% (n=217)). The mean age was 51.78 years (±10.24); male participants were slightly older (54.67 years (±11.52)) than females (51.29 years (±9.96)), although this difference was not statistically significant (P=0.0674). The majority were self-employed (85.11% (n=183)), and most were non-consumers of alcohol (88.97% (n=226)). Only 11.02% (n=28) consumed alcohol, and among them, 40.74% (n=11) were occasional consumers, with females comprising most of the occasional drinkers (90% (n=10)). Females had significantly higher BMI than males (women: 26.59 (±7.27), men: 23.29 (±5.12), P=0.008). However, males had higher abdominal obesity than females (women: 0.89 (±0.08), men: 0.97 (±0.07), P=0.0006). About 22.4% of participants had metabolic syndrome (Std. Err. 0.026, 95% CI: 0.281-0.3199). Middle-aged individuals had 3.55 times higher odds of hypertension compared to younger adults (OR = 3.55 (95% CI: 1.51-8.37), P=0.004), and older adults had 6.85 times higher odds compared to younger adults (OR = 6.85 (95% CI: 2.21-21.28), P=0.001). The study showed a crude prevalence of diabetes mellitus of 25.21% (n=30/119, 95% CI: 17.5%-34.4%), and the adjusted diabetes prevalence increased to 30.3% (95% CI: 24.7%-36.0%).

Conclusion

ASCVD risk factors were relatively high among PLWH. Screening for ASCVD should begin in middle-aged PLWH for early detection, and primary CVD preventive measures should be implemented to reduce the ASCVD burden.

## Introduction

For the past decade, sub-Saharan Africa (SSA) has seen a rise in atherosclerotic cardiovascular disease (ASCVD) [1]. The increasing burden of ASCVD in SSA is related to the epidemiological transition occurring in the region. The burden of non-communicable diseases (NCDs) is worsened by the rising numbers of infectious diseases such as HIV. The risk factors underpinning the high prevalence of ASCVD are both modifiable and non-modifiable [2-3]. Although non-modifiable factors such as age, sex, and race contribute to approximately 63%-80% of ASCVD prediction, controlling for modifiable factors leads to significant reductions in ASCVD [4]. Additionally, a unique contributor to ASCVD among people living with HIV (PLWH) is HIV infection-related factors. These include increased systemic inflammation, endothelial dysfunction, and lipodystrophy [5].

In a Kenyan study examining the association between inflammatory markers, HIV status, and traditional cardiovascular disease (CVD) risk factors, it was found that PLWH had a 51% higher IL-6 concentration, and higher mean IL-1β, tumour necrosis factor alpha (TNF-α), and high-sensitivity CRP (hsCRP) levels compared to HIV-negative individuals, independent of CVD risk factors [6]. The proliferation of IL-6 among PLWH is known to activate a cascade of arteriosclerosis by promoting the expression of cellular adhesion molecules, smooth muscle cell proliferation, leukocyte recruitment, and matrix degeneration, all of which contribute to plaque build-up in the arteries [7,8]. Elevated IL-6 has also been implicated in foam-cell formation within arterial walls, contributing to wall dysfunction and reduced arterial compliance [8]. Additionally, activation of these inflammatory cytokines promotes the production of hepatic CRP, a strong marker for ASCVD. Other inflammatory markers, such as TNF-α, have also been shown to increase vascular inflammation and promote plaque instability, thereby increasing ASCVD risk among individuals with HIV [6,8].

Beyond these pathophysiologic pathways, traditional risk factors common in non-HIV populations also influence ASCVD risk in PLWH and further exacerbate their risk. For instance, dyslipidemia, characterized by high low-density lipoprotein (LDL) cholesterol, triglycerides, and total cholesterol, with or without reduced high-density lipoprotein (HDL) cholesterol, is considered atherogenic and compromises vascular compliance [8]. Increased BMI in the obesity range, along with other traditional risk factors such as abdominal obesity, sedentary lifestyle, cigarette smoking, and high alcohol consumption, is also associated with ASCVD in both the general population and PLWH [3]. For these reasons, primary modifiable factors are usually the target of health education and promotion to reduce the risk of secondary modifiable factors such as hypertension, diabetes mellitus, and dyslipidemia [1,9]. The relevance of this approach is based on the fact that reducing primary modifiable factors decreases ASCVD risk. A high burden of ASCVD among affected individuals is likely to result in reduced life expectancy and diminished quality of life [10].

The role of alcohol consumption in ASCVD is diverse, and the relationship is not linear but rather J- or U-shaped, indicating an amount-dependent effect. It is known that moderate alcohol consumption is associated with a reduced risk of ASCVD, whereas binge consumption, on the contrary, is associated with an increased risk of ASCVD [11,12]. The mechanism is related to alcohol’s ability to activate the renin-angiotensin-aldosterone system (RAAS), leading to increased production of angiotensin II, a potent vasoconstrictor [13], as well as its direct cardiogenic effects [14] and contribution to endothelial dysfunction [8]. Its effect among PLWH of Black ethnicity, who bear the highest burden of ASCVD, is less explored.

Additionally, the intersection of HIV infection-related ASCVD factors, traditional factors, and lifestyle factors is often poorly defined, especially in resource-poor settings. In such settings, including some developing countries, ASCVD screening is ineffective and sometimes compromised by high patient-to-professional ratios, inadequate logistics for screening, and poor implementation of NCD policy frameworks.

It is therefore relevant for scientists to explore this grey area of intersection to guide policy formulation. The current study therefore sought to determine and characterize the risk factors of ASCVD among PLWH in the Ashanti Region.

## Materials and methods

Study setting, study design, and recruitment

Kumasi is the second-largest city in Ghana. It is a cosmopolitan city with multi-ethnic diversity. Komfo Anokye Teaching Hospital (KATH) is a teaching hospital serving the Ashanti Region and surrounding regions in the middle belt. In addition to serving as a teaching hospital, it has a polyclinic that also functions as a district hospital for greater Kumasi and nearby cities. KATH provides specialist care to both paediatric and adult patients, including an HIV clinic that serves both groups. In the Ashanti Region, almost all district hospitals, quasi-government facilities, and some Christian Health Association of Ghana (CHAG) hospitals also provide such services. In Kumasi, another facility that offers services to PLWH is the Kwame Nkrumah University of Science and Technology (KNUST) Hospital. It serves as a district hospital, receiving referrals from both government and private hospitals in the Oforikrom Municipality and other nearby districts. It has specialist care services, and the HIV clinic is one of them, serving PLWH.

Sample and sampling technique

This was a hospital-based cross-sectional study that spanned from September 2023 to September 2024. A total sample size of 254 participants was recruited for the study. Sample size estimation was based on the calculation below.

Based on a 95% CI and a 5% allowable margin of error, the sample size was calculated using the equation below [15]:

\begin{equation} n = \frac{Z^2 \cdot p \cdot (1-p)}{e^2} \end{equation}

Where: Z = the value corresponding to the desired level of confidence. The standard score for a 95% CI is 1.96.

P = an estimate of the prevalence of alcohol use among PLWH, taken as 18% based on the prevalence reported in a WHO/ISH community-based study on moderate and high ASCVD risk [16].

E = allowable margin of error, which is 5%.

The sample size is calculated as:

\[ n = \frac{Z^2 \cdot p \cdot (1 - p)}{E^2} \]

Substituting the values: \begin{align*} n &= \frac{(1.96)^2 \times 0.18 \times (1 - 0.18)}{(0.05)^2} \\ &= \frac{3.8416 \times 0.18 \times 0.82}{0.0025} \\ &= \frac{0.691488 \times 0.82}{0.0025} \\ &= \frac{0.56702016}{0.0025} \\ &= 226.808064 \end{align*}

Rounding up to the nearest whole number, the required sample size is:

\[ \boxed{227} \]

 ≈ 227 participants

Using an attrition rate of 15%, a total of 260 cases were recruited.

KATH: 80% of 260 = 208

KNUST Hospital: 52

Participants were PLWH who attended the KATH and KNUST HIV clinics for care. To minimize inconvenience, recruitment was conducted on clinic days. Participants were selected through systematic sampling, where every third person in the queue to receive care was approached. Upon agreeing to participate in the study, written consent was obtained. Questionnaires were administered through interviews by trained research assistants, who were mostly nurses and peer educators at the HIV clinics, under the supervision of the principal investigator. Interviews were conducted in the local language (Twi), and in Hausa for those who did not speak Twi. This approach helped minimize the risk of participants misunderstanding the questions or interpreting them differently from the interviewer.

Field and laboratory procedures

Participants in the queue to receive care were sampled using a systematic approach. Every third person was contacted, and those who consented were asked about their last meal; individuals who had eaten that morning were not enrolled. Blood sampling was performed early in the morning, between 6:00 am and 10:00 am, by a trained phlebotomist from KATH and a professional laboratory technician from KNUST Hospital. At least 3-5 mL of whole blood was collected from each participant.

After sampling, the samples were placed in a test tube rack until all collections were completed. Whole blood samples for HbA1c analysis were collected in EDTA tubes, while serum separator tubes (SST) were used for lipid profile analysis. A total of 119 samples were randomly selected for both serum lipid and HbA1c measurement.

HbA1c measurements were performed on the same day of sampling. Serum tubes were centrifuged and stored at -80 °C until further analysis. Centrifugation was conducted using a cooling centrifuge (ACNA 90-1 Centrifuge, Jiangsu Zhengji, China) at 3500 RPM to ensure sample integrity.

Serum obtained after centrifugation was transferred into two separate Eppendorf tubes labelled A and B and stored at -80 °C until analysis. A facility-based stadiometer was used to measure weight and height, and a tape measure was used to measure waist and hip circumference following the WHO STEPS protocol. Blood pressure (BP) was measured three times while participants were seated in an armrest chair with the arm supported on a table. BP readings were taken using the OMORN 10 Series Upper Arm Blood Pressure Monitor (Omron Healthcare Inc., Japan) with an adult-size HEM-FL31-B cuff (9"-17" / 22-42 cm).

Inclusion and exclusion criteria

Participants who were Ghanaians and ≥18 years, had been confirmed HIV-positive, and visited the study sites for medical care were included in the study. Participants who were HIV-positive but critically ill were excluded. Because weight and height were needed to determine BMI, potential participants who were amputated or had fractures were also excluded. Pregnant and breastfeeding women were also excluded.

Study variables, measurements, and their definitions

Measurement

Outcome variables: The primary outcome measures of interest were the prevalence of ASCVD risk factors, which included hypertension, high cholesterol, diabetes mellitus, tobacco smoking, and alcohol consumption. These risk factors were chosen a priori. For hypertension, three BP measurements were taken at 5-minute intervals. The average of the three BP readings was used. Participants were categorized as having high BP if the average systolic BP (SBP) was ≥140 mmHg and/or the average diastolic BP (DBP) was ≥90 mmHg, according to standard staging guidelines [17]. For diabetes mellitus, HbA1c measurements ≥6.5% were considered diagnostic of diabetes, and those <5.7% were considered non-diabetic. A fasting lipid profile was defined as high if total cholesterol was ≥6.22 mmol/L (240 mg/dL), LDL ≥4.14 mmol/L (160 mg/dL), and triglycerides ≥1.70 mmol/L (150 mg/dL) [18,19]. LDL was estimated using the formula:

LDL cholesterol = total cholesterol - HDL cholesterol - (triglycerides ÷ 5).

Active tobacco smoking was defined as self-reported cigarette, pipe, or cigar smoking within the past 30 days. Alcohol consumption was also assessed using the AUDIT-C. Alcohol consumption patterns were evaluated based on questions regarding the frequency and serving amounts of alcoholic beverages (i.e., beer, wine, and spirits) consumed in the preceding month. Standard serving amounts were defined as 1 bottle (0.5 L) of beer, 0.25 L of wine (including palm wine), and 8 cL of spirits (including spirits traditionally consumed in Ghana such as brukutu, pito, and akpeteshie). Responses to these questions were used to categorize participants as consumers within limits or non-consumers. Alcohol consumption was categorized into three groups: non-consumers, consumers within limits (moderate consumers), and consumers beyond limits (heavy consumers). Non-consumers were participants who had never consumed alcohol in their lifetime. Moderate consumers were defined as consuming up to 21 units of alcohol per week for men and 14 units per week for women. Heavy consumers were those who consumed amounts above the moderate threshold for both men and women. The biological risk factors were selected a priori based on existing studies and included anthropometric measurements such as weight, height, and waist circumference. Formulas for BMI and waist-to-hip ratio (WHR) have already been described in our previous publication [2]. Metabolic syndrome (MetS) was defined according to the harmonized Joint Interim Statement criteria, with slight modifications to suit the characteristics of our data. MetS was identified as the presence of ≥3 of the following: obesity (BMI ≥30 kg/m²), high triglycerides (≥1.7 mmol/L), low HDL cholesterol (<1.0 mmol/L in men or <1.3 mmol/L in women), elevated BP (systolic ≥130 mmHg or diastolic ≥85 mmHg), and elevated fasting glucose (diabetes diagnosis).

*MetS* *Assessment*

In this study, we defined MetS according to the harmonized Joint Interim Statement criteria (Alberti et al., 2009) [20], which require ≥3 of the following: central obesity (waist circumference >102 cm in men or >88 cm in women), hypertriglyceridemia (fasting triglycerides ≥1.7 mmol/L), low HDL cholesterol (<1.0 mmol/L in men or <1.3 mmol/L in women), elevated BP (systolic ≥130 mmHg or diastolic ≥85 mmHg), and elevated fasting glucose (HbA1c ≥5.6%). Participants with more than two missing components were excluded from classification. MetS scoring required complete data for all components, and participants were classified as having MetS if ≥3 components were present. Severity was categorized as: none (0-2 components), mild (3), moderate (4), or severe (5). The 95% CIs for MetS prevalence and the distribution of components were calculated using the exact binomial method (Clopper-Pearson interval), which is the gold standard for small-to-moderate sample sizes and provides conservative estimates that guarantee 95% coverage probability, making it appropriate for proportions in biomedical research such as this study.

Statistical analysis

Primary data were gathered using REDCap. The data were extracted into Microsoft Excel (version 2013), and data cleaning was performed in Microsoft Excel before being imported into STATA/SE version 14.2 for statistical analysis. Normality was tested, and descriptive analyses such as means (SDs), frequencies, percentages, and tables were used to describe the study population. The IQR was used for variables that were not normally distributed (SBP, DBP, WHR, BMI, HbA1c, lipid profile, alcohol consumption (Appendix 1). Non-normality was confirmed using the Shapiro-Wilk test (for n < 5000) and the Skewness/Kurtosis tests. Using the Mann-Whitney test, null hypotheses were tested for selected variables, and p-values <0.05 were considered statistically significant. Chi-square and Fisher’s exact tests were used to determine differences in the categorical ASCVD risk outcomes (gender, alcohol categorization, BMI categorization, hypertension, diabetes, dyslipidemias, and smoking categorization).

The exact binomial method was used to establish the CI. To estimate the crude prevalence, the formula below was used: 

\[ \text{Crude prevalence} = \left( \frac{\text{Cases}}{\text{Total}} \right) \times 100 \]

To calculate the WHO population weight for each age group, we adopted the WHO-recommended standard weights as follows: <40 years: 0.2907, 40-49 years: 0.1734, 50-59 years: 0.1546, 60-69 years: 0.1331, and ≥70 years: 0.2482.

The adjusted age prevalence was estimated as:

\[ P_{\text{adjust}} = \sum (\text{Prevalence} \times \text{WHO Weight}) \]

The 95% CI for the adjusted age-specific estimates was calculated using bootstrap resampling (1,000 replicates) and the Fay & Feuer method, accounting for clustering by age group.

Alternative estimates were generated using the svyset command in Stata version 14.2 with strata-specific weights. Sensitivity analysis was performed by comparing results using the WHO 2000-2025 standard population versus the Segi world standard population, and small subgroups such as participants aged above 70 years were excluded.

## Results

Background of respondents

The mean age of participants was 51.78 years (SD = 10.24). Males were older than females, but the difference was not statistically significant (males: 54.67 years, SD = 11.52; females: 51.29 years, SD = 9.96; P = 0.0674). Most participants, 48.2% (n = 121), had completed either secondary school or vocational school and were self-employed (72.9%, n = 183). The majority, 72.5% (n = 150), earned less than 500 cedis per month (Table 1).

**Table 1:** Background distribution of participants. The results are presented as n = frequency, % = percentage, SD = standard deviation, and IQR = interquartile range. Statistical significance was set at P < 0.05. To test for differences in age between males and females, an independent samples t-test was used, which showed a non-significant age difference between groups (t(247) = 1.84, P = 0.067). However, the effect size suggested a small to moderate difference (Cohen’s d = 0.33, 95% CI: -0.02 to 0.69), with males being approximately 3.4 years older on average than females. To test for differences in the number of children and income between male and female participants, the Wilcoxon rank-sum test was used because the data showed non-normal distribution. Males (n = 25) reported significantly more children (Median = 4, IQR = 2-5) compared to females (n = 199; Median = 3, IQR = 2-4). The effect size indicated a medium effect (Cohen’s d = 0.50, 95% CI: 0.08 to 0.92). A highly significant difference in income was also observed between males and females (Wilcoxon z = 4.52, P < 0.0001). The effect size was large (Cohen’s d = 0.93, 95% CI: 0.53 to 1.33), indicating that males had substantially higher income than females. Approximately 9.7% of the variance in income was explained by gender.

Variable	Female, n (%)	Male, n (%)	Total n (%)	t / z-value	p-value
Age (mean ± SD)	51.29 (9.96)	54.67 (11.52)	51.78 (10.24)	1.8373 (t)	0.0674
Age categorisation					
Less than 25	2 (100.00)	0 (0.00)	2 (100.00)	-	-
26-45 years	56 (91.80)	5 (8.20)	61 (100.00)	-	-
46-65 years	137 (84.57)	25 (15.43)	162 (100.00)	-	-
Above 65 years	18 (75.00)	6 (25.00)	24 (100.00)	-	-
Total	213 (85.54)	36 (14.46)	249 (100.00)	-	-
Education					
No formal education	43 (87.76)	6 (12.24)	49 (100.00)	-	-
Basic education complete	21 (87.50)	3 (12.50)	24 (100.00)	-	-
Basic education incomplete	33 (94.29)	2 (5.71)	35 (100.00)	-	-
Secondary/vocational complete	106 (87.60)	15 (12.40)	121 (100.00)	-	-
Secondary/vocational incomplete	7 (43.75)	9 (56.25)	16 (100.00)	-	-
Tertiary	5 (83.33)	1 (16.67)	6 (100.00)	-	-
Total	215 (85.66)	36 (14.34)	251 (100.00)	-	-
Marital status					
Never married	28 (84.85)	5 (15.15)	33 (100.00)	-	-
Married/cohabiting	69 (75.82)	22 (24.18)	91 (100.00)	-	-
Divorced/separated	47 (90.38)	5 (9.62)	52 (100.00)	-	-
Widow/widower	71 (94.67)	4 (5.33)	75 (100.00)	-	-
Total	215 (85.66)	36 (14.34)	251 (100.00)	-	-
Occupation					
Employee	13 (59.09)	9 (40.91)	22 (100.00)	-	-
Self-employed	164 (89.62)	19 (10.38)	183 (100.00)	-	-
Not employed	38 (86.36)	6 (13.64)	44 (100.00)	-	-
Other	0 (0.00)	2 (100.00)	2 (100.00)	-	-
Total	215 (85.66)	36 (14.34)	251 (100.00)	-	-
Number of children (IQR)	3 (2-4)	4 (2-5)	3 (2-4)	1.928	0.0538
1-3 children	138 (93.88)	9 (6.12)	147 (100.00)	-	-
4-6 children	55 (82.09)	12 (17.91)	67 (100.00)	-	-
Above 7 children	6 (60.00)	4 (40.00)	10 (100.00)	-	-
Total	199 (88.84)	25 (11.16)	224 (100.00)	-	-
Income per month (IQR)	200 (50-500)	950 (500-1500)	200 (50-600)	4.516 (z)	0.0001
<500	138 (92.00)	12 (8.00)	150 (100.00)	-	-
500-1000	28 (80.00)	7 (20.00)	35 (100.00)	-	-
1000-1500	3 (42.86)	4 (57.14)	7 (100.00)	-	-
>1500	8 (53.33)	7 (46.67)	15 (100.00)	-	-
Total	177 (85.51)	30 (14.49)	207 (100.00)	-	-

Among current alcohol consumers, 40.7% (n = 11) were occasional consumers, and beer was the preferred beverage (59%, n = 16), as shown in Table 2. Of the total participants, 7.48% (n = 19) consumed alcohol within recommended limits, whereas 3.5% (n = 9) consumed alcohol beyond recommended limits (Table 2).

**Table 2:** Lifestyle history of participants. The results are presented as n = frequency, % = percentage, and IQR. A significant difference in current alcohol consumption was observed between males and females (Wilcoxon z = 3.53, p = 0.0004). The effect size was medium-to-large (Cohen's d = 0.78, 95% CI: 0.43 to 1.14), indicating that males consumed substantially more alcohol than females. Approximately 7.2% of the variance in alcohol consumption was explained by gender. However, for former alcohol consumption, there were no statistically significant differences between gender groups, whether measured in units (Wilcoxon z = -0.59, p = 0.553) or in grams (Wilcoxon z = -0.58, p = 0.561). Effect sizes were negligible (Cohen's d = -0.035 for grams consumed), with CIs including zero.

Variable	Female, n (%)	Male, n (%)	Total n (%)	z-value	p-value
Current alcohol consumption (IQR)	0 (0-0)	0 (0-13.6)	0 (0-13.6)	3.533	0.0004
Alcohol consumption pattern among current consumers					
Daily	1 (12.50)	7 (87.50)	8 (100.00)		
2-3 times per week	4 (100.00)	0 (0.00)	4 (100.00)		
Once a week	2 (50.00)	2 (50.00)	4 (100.00)		
Occasional	10 (90.91)	1 (9.09)	11 (100.00)		
Total	17 (62.96)	10 (37.04)	27 (100.00)		
Drink categorization among current consumers					
Beer	12 (75.00)	4 (25.00)	16 (100.00)		
Spirit - Exotic	0 (0.00)	2 (100.00)	2 (100.00)		
Wine	3 (75.00)	1 (25.00)	4 (100.00)		
Apeteshie	1 (25.00)	3 (75.00)	4 (100.00)		
Palm wine	1 (100.00)	0 (0.00)	1 (100.00)		
Pito	-	-	-		
Total	17 (62.96)	10 (37.04)	27 (100.00)		
Drink categorization among former consumers					
Beer	33 (86.84)	5 (13.16)	38 (100.00)		
Spirit - Exotic	1 (50.00)	1 (50.00)	2 (100.00)		
Wine	9 (90.00)	1 (10.00)	10 (100.00)		
Apeteshie	-	-	-		
Palm wine	-	-	-		
Pito	-	-	-		
Total	43 (86.00)	7 (14.00)	50 (100.00)		
Alcohol consumption categorization among current consumers					
Non-consumers	199 (88.05)	27 (11.95)	226 (100.00)		
Moderate consumers	14 (73.68)	5 (26.32)	19 (100.00)		
Heavy consumers	4 (44.44)	5 (55.56)	9 (100.00)		
Total	217 (85.43)	37 (14.57)	254 (100.00)		
Alcohol consumption categorization among former consumers					
N (IQR)	0 (0-0)	0 (0-0)	0.83 (2.17)	-0.593	0.553
Non-consumers	172 (84.73)	31 (15.27)	203 (100.00)	-	-
Moderate consumers	44 (88.00)	6 (12.00)	50 (100.00)	-	-
Heavy consumers	1 (100.00)	0 (0.00)	1 (100.00)	-	-
Total	217 (85.43)	37 (14.57)	254 (100.00)	-	-
History of smoking					
Former smokers	0 (0.00)	0 (0.00)	0 (0.00)		
Current smokers	0 (0.00)	2 (100.00)	2 (100.00)		

The crude prevalence of hypertension was 27.67% (n = 70), and it was more prevalent among women, 85.7% (n = 60), than men, 14.3% (n = 10). Males had a higher pulse pressure (PP) (IQR = 50 (4057)) than females (IQR = 43 (36-53)), and this difference was statistically significant (P = 0.0106). The crude prevalence of diabetes was 25.21% (n = 30), with females accounting for 90.0% (n = 27) and males 10.0% (n = 3). Females had a higher BMI (IQR = 25 (22-30)) compared to males (IQR = 22 (20-26)), and this was statistically significant (P = 0.0012). Conversely, males had higher abdominal obesity (IQR = 1.0 (0.9-1.0)) compared to females (IQR = 0.9 (0.8-0.9)) (P = 0.0004) (Table 3).

**Table 3:** Baseline cardiovascular profile of study participants. The results are presented as n = frequency, % = percentage, SD, and IQR. The t-test was used to compare statistical differences between normally distributed numerical variables, while the Mann-Whitney U test was used to assess statistical significance for non-normally distributed variables. A p-value of <0.05 was considered statistically significant. LDL and total cholesterol (TC) were normally distributed; therefore, a t-test was used to test the hypothesis that LDL does not differ by gender. LDL: Low-density lipoprotein.

Variable	Female, n (%)	Male, n (%)	Total n (%)	t / z-value	p-value
Systolic BP (IQR)	121 (110-136)	124 (118-140)	122 (111-138)	1.542 (z)	0.1232
Diastolic BP (IQR)	80 (72-86)	78 (71-84)	79 (72-86)	-0.761 (z)	0.4466
Proportion of hypertension					
Non-hypertensive	156 (85.25)	27 (14.75)	183 (100.00)		
Hypertensive	60 (85.71)	10 (14.29)	70 (100.00)		
Blood pressure-related indices					
Mean arterial pressure (IQR)	93 (85-103)	95 (88-105)	93 (86-103)	0.445	0.6564
Pulse pressure (IQR)	43 (36-53)	50 (40-57)	44 (36-53)	2.557	0.0106
HbA1c (IQR)	5.5 (5.0-6.5)	5.6 (5.2-6.0)	5.5 (5.0-6.5)	0.305	0.7604
Proportion with diabetes					
Persons without diabetes	73 (82.02)	16 (17.98)	89 (100.00)		
Persons with diabetes	27 (90.00)	3 (10.00)	30 (100.00)		
Total	100 (84.03)	19 (15.97)	119 (100.00)		
Serum lipids					
Total cholesterol, mean (±SD)	4.38 (1.132)	4.07 (1.028)	4.33 (1.117)	-1.1392 (t)	0.2569
LDL cholesterol (mmol/L), mean (±SD)	2.67 (0.987)	2.39 (0.944)	2.62 (0.981)	-1.1521 (t)	0.2517
HDL cholesterol (IQR)	1.1 (1.0-1.4)	1.2 (1.0-1.5)	1.1 (1.0-1.4)	0.703 (z)	0.482
Triglycerides (IQR)	1.0 (0.7-1.4)	0.8 (0.7-1.0)	0.9 (0.7-1.4)	-1.844 (z)	0.0651
Anthropometry					
BMI (IQR)	25 (22-30)	22 (20-26)	25 (22-30)	-3.233 (z)	0.0012
Waist-to-hip ratio (IQR)	0.9 (0.8-0.9)	1.0 (0.9-1.0)	0.9 (0.86-0.9)	3.547 (z)	0.0004
Viral load					
Viral load (copies/mL)	0 (0-22.5)	20 (0-56.3)	0 (0-22.5)	1.900 (z)	0.0574
ART treatment history					
<2006	34 (89.47)	4 (10.53)	38 (100.00)		
2007-2012	52 (86.67)	8 (13.33)	60 (100.00)		
2013-2018	53 (84.13)	10 (15.87)	63 (100.00)		
2019-2024	76 (84.44)	14 (15.56)	90 (100.00)		
Total	215 (85.66)	36 (14.34)	251 (100.00)		

**Figure 1:**
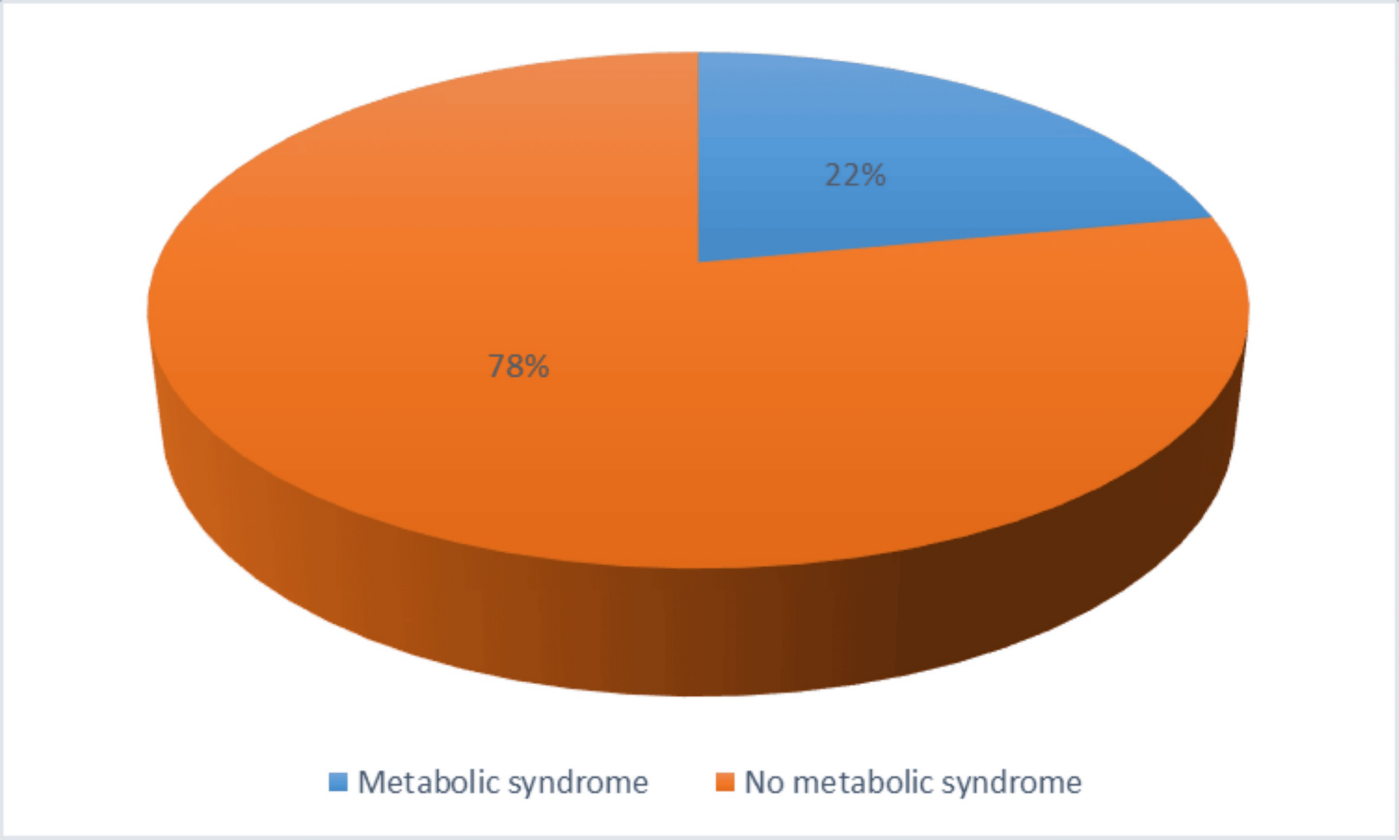
Prevalence of metabolic syndrome among participants. Prevalence of metabolic syndrome was 22% (57/254), with a 95% CI of 17.46-28.07% (exact binomial). IIn the sensitivity analysis for metabolic syndrome, inverse probability weighting was applied to account for missing data. The weighted prevalence was 12.4% (95% CI: 5.4-25.7%), which was higher than the complete-case estimate of 5%.

Among PLWH, the crude prevalence of hypertension was 27.67% (n = 70). Hypertensive participants had a higher mean systolic BP (150.68 mmHg ± 16.51) compared to non-hypertensives (115.46 mmHg ± 12.38), with an overall mean of 125.20 mmHg (± 20.84), and this difference was statistically significant. The prevalence of any form of dyslipidaemia was 23.6% (95% CI: 0.185-0.293, P = 0.036). The prevalence of obesity among participants was 24.0%, and the prevalence of metabolic syndrome was 22.4% (95% CI: 0.175-0.281), as shown in Figure 1 and Table 4.

**Table 4:** ASCVD risk factors and their proportions. The results are presented as n = frequency, % = percentage, SD, IQR, and standard error. Statistical significance was set at p < 0.05. A t-test was used to compare numerical variables, while a chi-square test was used to assess statistical significance between categorical variables. B/P: Blood pressure; PP: Pulse pressure; MAP: Mean arterial pressure; ASCVD: Atherosclerotic cardiovascular disease; LDL: Low-density lipoprotein; HDL: High-density lipoprotein.

Risk Factor	Category / Variable	Obs	Proportion	Std.Err	95% CI (Lower-Upper)	Mean (±SD)
Diabetes	Persons without diabetes (<6.5%)	119	0.748	0.04	0.660-0.823	5.22 (0.61)
	Persons with diabetes (≥6.5%)	119	0.252	0.04	0.177-0.340	9.16 (2.79)
Hypertension - Non-hypertensive	Non-hypertensive	253	0.723	0.028	0.664-0.778	-
	Systolic BP	183	-	-	-	115.46 (12.38)
	Diastolic BP	183	-	-	-	74.46 (7.77)
	Pulse pressure (PP)	183	-	-	-	41.00 (9.63)
	Mean arterial pressure (MAP)	183	-	-	-	88.12 (8.41)
Hypertension - Hypertensive	Hypertensive	253	0.276	0.028	0.222-0.336	-
	Systolic BP	70	-	-	-	150.68 (16.51)
	Diastolic BP	70	-	-	-	92.41 (9.25)
	Pulse pressure (PP)	70	-	-	-	58.27 (15.89)
	Mean arterial pressure (MAP)	70	-	-	-	111.83 (9.58)
Dyslipidemia - Absent	Absent	254	0.764	0.027	0.707-0.815	-
	Total cholesterol	59	-	-	-	4.11 (4.54)
	LDL-cholesterol	58	-	-	-	2.38 (0.63)
	HDL-cholesterol	58	-	-	-	1.36 (0.26)
	Triglycerides	59	-	-	-	0.85 (0.28)
Dyslipidemia - Present	Present	254	0.236	0.027	0.185-0.293	-
	Total cholesterol	60	-	-	-	4.54 (1.39)
	LDL-cholesterol	58	-	-	-	2.78 (1.09)
	HDL-cholesterol	60	-	-	-	1.04 (0.43)
	Triglycerides	60	-	-	-	1.39 (0.69)
BMI Category	Underweight	254	0.059	0.015	0.033-0.096	16.29 (1.91)
	Normal	254	0.437	0.031	0.375-0.500	22.07 (1.75)
	Overweight	254	0.264	0.028	0.211-0.323	27.12 (1.36)
	Obese	254	0.24	0.027	0.189-0.298	34.74 (8.48)
Metabolic Syndrome	Absent	254	0.776	0.026	0.719-0.825	-0.2937
	Present	254	0.224	0.026	0.175-0.281	0.3199
Alcohol Consumption	Non-consumers	254	0.89	0.02	0.845-0.925	0
	Consumption within limits	254	0.075	0.017	0.046-0.114	12.93 (2.99)
	Heavy consumption	254	0.035	0.012	0.016-0.066	38.80 (17.40)

The study showed that males had 15.9% lower odds of hypertension compared to females, but the association was not statistically significant (OR = 0.841, 95% CI: 0.373-1.897, P = 0.676). Middle-aged individuals had 3.55 times higher odds of hypertension compared to young adults (OR = 3.55, 95% CI: 1.51-8.37, P = 0.004). Older adults had 6.85 times higher odds of hypertension compared to young adults (OR = 6.85, 95% CI: 2.21-21.28, P = 0.001), as shown in Table 5.

**Table 5:** Factors associated with hypertension: adjusted odds ratios for sex and age categories among study participants (N = 248). Model characteristics: Observations: 248 Pseudo R²: 0.050 Model p-value: 0.002 ***p < 0.01 The results are presented as Coef = coefficient, t-value = t-statistic, SD, IQR, and standard error. A t-statistic was used to assess statistical significance in the regression analysis, and a p-value < 0.05 was considered statistically significant. The level of significance is indicated by *.

Predictor	Odds Ratio	Std. Error	p-value	95% CI	Sig.
Gender (Ref: Female)	-	-	-	-	-
Male	0.841	0.349	0.676	0.373-1.897	-
Age (Ref: Young)	-	-	-	-	-
Middle age	3.553	1.553	0.004	1.509-8.367	***
Old age	6.85	3.961	0.001	2.205-21.279	***

The crude prevalence of diabetes mellitus was 25.21% (n = 30/119, 95% CI: 17.5%-34.4%). After age-standardization using WHO World Standard Population weights, the adjusted diabetes prevalence increased to 30.3% (95% CI: 24.7%-36.0%), reflecting the younger age structure of the study population compared to the reference standard.

As shown in Table 6, the prevalence of diabetes mellitus increased with age, with HbA1c ≥6.5% rising sharply from 10.7% among participants under 40 years to 41.7% in those above 70 years. Participants above 70 years had the highest prevalence of diabetes (41.7%) and contributed most to the age adjustment (10.3%) due to their larger weight in the standard population. Participants aged 50-59 years accounted for the greatest absolute burden (37.0% prevalence; 34 cases). Age-adjustment increased the overall prevalence of diabetes by 4.1 percentage points.

**Table 6:** Age-specific and age-standardized diabetes prevalence (N = 249). Age-standardization was performed using the WHO World Standard Population weights. The age-standardized prevalence was 30.3% (95% CI: 24.7-36.0%). Adj: Adjusted.

Age Group	Patients	Cases	Prevalence (%)	WHO Weight	Weighted Contribution
<40	28	3	10.7	0.291	0.031 (3.1%)
40-49	74	22	29.7	0.173	0.052 (5.2%)
50-59	92	34	37	0.155	0.057 (5.7%)
60-69	43	16	37.2	0.133	0.050 (5.0%)
≥70	12	5	41.7	0.248	0.103 (10.3%)
Total	249	80	Crude: 25.2%	1	Adj: 29.3%

## Discussion

This study aimed to assess the prevalence of risk factors for ASCVD among PLWH receiving care at two treatment centers in Kumasi, Ashanti Region, Ghana. Recruitment was carried out among participants who were not known to be living with hypertension or diabetes. Overall, the study showed a high prevalence of risk factors among PLWH.

The study found a significant difference in alcohol consumption between sexes, with males consuming significantly higher amounts of alcohol than females. In Africa, including Ghana, alcohol consumption is often considered a masculine activity due to prevailing societal norms. In some societies, alcohol consumption is also viewed as a marker of social status. Women usually consume alcohol occasionally, as reflected in this study. Although men had a higher rate of alcohol consumption, their intake remained within the recommended limits.

The crude prevalence of hypertension in this study was 27.67% (n=70), which was lower than the findings of Lartey M et al. (2024) [21], who reported a significantly higher prevalence of hypertension among PLWH receiving dolutegravir (DTG).

The results were, however, comparable to a study conducted in the same setting by Sarfo FS et al. (2019), which reported hypertension prevalence of 23.4% among HAART users compared to 36.9% among HAART-naïve patients [22]. Although age variations existed among participants across studies, the high prevalence of hypertension observed remains a crucial call to action for reducing ASCVD risk among PLWH.

In the current study, males had 15.9% lower odds of hypertension compared to females, although the association was not statistically significant (OR = 0.841, 95% CI: 0.373-1.897, P = 0.676). This suggests that sex alone is not a major determinant of hypertension risk in PLWH. Middle-aged individuals had 3.55 times higher odds of hypertension compared to younger individuals (OR = 3.55, 95% CI: 1.51-8.37, P = 0.004), indicating that hypertension risk rises substantially in midlife. Screening and preventive strategies should therefore be targeted toward this age group. Older adults had 6.85 times higher odds of hypertension compared to younger individuals (OR = 6.85, 95% CI: 2.21-21.28, P = 0.001), suggesting that increasing age is a strong, independent risk factor for hypertension. Hypertension among PLWH is due to several factors, including the side effects of certain HAART medications, such as DTG [23-26]. Recent studies have observed that DTG is associated with hypertension [25,26]. Another significant factor in the etiology of hypertension among PLWH is the vascular dysfunction associated with HIV infection. There are several pathophysiological pathways relating to this aetiology, including an increased systemic inflammatory threshold and the induction of endothelial dysfunction. The proliferation of IL-6 among PLWH is known to activate a cascade of arteriosclerotic processes by promoting the expression of cellular adhesion molecules, smooth muscle cell proliferation, leukocyte recruitment, and matrix degeneration, all of which contribute to the buildup of plaque in the arteries [6]. Another factor contributing to undiagnosed and uncontrolled hypertension in this cohort is the competing priorities of health care professionals. Reducing viral load is critically important, and health care workers may focus on this more than on screening for hypertension. To minimize the risk of hypertension, health care workers need to prioritize ASCVD prevention alongside viral suppression, as ASCVD often leads to earlier mortality in PLWH, even before complications from increasing viral load. Although ageing plays a significant role in the aetiology of hypertension among PLWH, ageing is non-modifiable. However, modifiable risk factors that can potentially exacerbate the process can be targeted through health education and CVD prevention. Strategies such as increasing adherence to the Mediterranean diet, discouraging heavy alcohol consumption, and promoting smoking cessation should be encouraged. Health care workers, as well as government agencies, have roles to play in advancing these strategies. Although health workers provide health education during clinic visits, encouraging behavioural change remains crucial in reducing risk. Screening for secondary risk factors like hypertension should be consciously carried out during clinic visits.

Our analysis also showed significant differences in income between men and women. Many of those living below the poverty line in the study were women. In the African context, men are traditionally expected to be the head of the household and the primary breadwinner; therefore, many men diversify their income sources, giving them an economic advantage over women. Higher income is associated with poor eating habits, such as increased consumption of sweetened and high-calorie foods, and when coupled with a sedentary lifestyle, this places individuals at increased risk of ASCVD [27]. Affluent men working in demanding jobs are also exposed to work-related stress, which raises serum cortisol levels and exposes them to ASCVD [28].

The study also observed that diabetes prevalence was relatively high. The analysis showed that the crude prevalence of diabetes (HbA1c ≥6.5%) was 25.21% (n=30; 95% CI: 17.5%-34.4%), and the group without diabetes (HbA1c <6.5%) accounted for 74.79% (n=89) of the sample. It was observed that, at 95% confidence, the true age-standardized prevalence lies between 24.7% and 36.0%. The wide confidence interval reflects uncertainty, likely due to the small sample size in the older age groups (particularly those aged ≥70 years, with only 12 patients). The design factor of the analysis properly accounted for stratification across the five age groups, finite population correction (population size = 43.9), and the survey weights (WHO standard weights) used in the analysis. Again, type 2 diabetes usually develops in older people (over the age of 65 years) and is affected by lifestyle and environmental factors, but is mainly driven by age-related decline in pancreatic function.

Although the classification of diabetes was based solely on HbA1c using IDF criteria, this may have overestimated or underestimated the true prevalence, especially because no confirmatory test was done, as was the case in this study. The observed prevalence of 25.21% is higher than that reported previously in one of the study settings [29], and this is possibly due to the older age distribution of participants. The high prevalence suggests that diabetes is indeed a major health concern among PLWH, particularly among those aged ≥70 years, who showed the highest prevalence (41.7%). This highlights an opportunity for health professionals to strengthen geriatric diabetes management and to screen adults younger than 40 years at even lower BMI thresholds. Individuals aged 40-50 years should be targeted for specific prevention programmes, and future research should investigate genetic and environmental drivers among PLWH. Last but not least, our study showed a metabolic syndrome prevalence of 22.44% among participants (95% CI: 17.46-28.07%). This is considerably lower than the 57.3% reported by Dzudzor B et al. (2023) among PLWH receiving HAART [30]. The 22.44% prevalence observed in our study likely reflects the younger cohort.

Limitations

The small sample size, especially in the older age groups, limits the precision of estimates and reduces generalizability. Additionally, the cross-sectional design limits causal inference. Another acknowledged limitation relates to the diagnosis of hypertension and diabetes mellitus. Clinically, hypertension is diagnosed over at least three visits, and diabetes is diagnosed with HbA1c on the first visit and a follow-up test. However, this does not affect our analysis, as the definitions used are widely accepted for epidemiological studies. Our analysis also did not account for the physical activity levels or dietary preferences of participants, which could influence the observed risk factors.

## Conclusions

In summary, hypertension risk increases dramatically with age, but sex does not appear to be a major factor in this population. Clinicians should therefore recognize that every HIV clinic visit is an opportunity to prevent ASCVD. Clinicians should focus on age-appropriate screening and prevention strategies, particularly for adults over 40 years. Hospitals should also be encouraged to use electronic health record systems that flag middle-aged and older adults for hypertension screening. In addition, hospitals can partner with NGOs to organize hypertension screening drives. For age-specific interventions, because early HAART initiation exposes young adults (18-39 years) to lifelong treatment, BP screening should be tied to viral load visits. Lapses in the early detection of these risks represent missed opportunities for health providers to intensify screening and reduce the long-term risk of ASCVD.

## Appendices

Appendix 1 

**Table 7:** Participant category of HAART usage. 3TC: Lamivudine; EFV: Efavirenz; DTG: Dolutegravir; TDF: Tenofovir disoproxil; LPV/r: Ritonavir-boosted lopinavir; ABC: Abacavir; CBV: Combivir; HAART: Highly Active Antiretroviral Therapy.

ART Regimen	Freq.	Percent (%)
3TC, EFV, DTG	1	0.4
3TC, EFV, TDF	5	1.99
3TC, TDF	1	0.4
3TC, TDF, DTG	203	80.88
3TC, TDF, LPV/r	1	0.4
ABC, DTG	4	1.59
ABC, TDF	3	1.2
ABC, TDF, DTG	2	0.8
ATV, TDF	2	0.8
CBV	1	0.4
DTG	1	0.4
EFV, DTG	2	0.8
EFV, TDF	18	7.17
TDF	4	1.59
TDF, LPV/r	3	1.2
Total	251	100
